# Correlation of sperm motility, acrosome integrity, protamine deficiency, and DNA fragmentation in proven and unproven Friesian Holstein bulls

**DOI:** 10.5455/javar.2024.k831

**Published:** 2024-09-30

**Authors:** Ristika Handarini, Abdullah Baharun, Annisa Rahmi, Deden Sudrajat, Anggraeni Anggraeni, Nurcholis Nurcholis, Hikmayani Iskandar, Tulus Maulana, Ekayanti Mulyawati Kaiin, Saiful Anwar, Syahruddin Said

**Affiliations:** 1 Department of Animals Science, Faculty of Agriculture, Djuanda University, Bogor, Indonesia; 2 Department of Animals Husbandry, Musamus University, Marauke, Indonesia; 3 Research Center for Animal Husbandry, National Research and Innovation Agency, Bogor, Indonesia; 4 Research Center for Applied Zoology, National Research and Innovation Agency, Bogor, Indonesia

**Keywords:** Dairy bull, frozen semen, molecular analysis, sperm quality

## Abstract

**Objective:**

The evaluation of frozen semen quality is an essential aspect in determining male fertility for artificial insemination programs. This study aims to evaluate the characteristics of Friesian Holstein (FH) bull-frozen semen in different classes (proven and unproven) based on protein profiling and molecular evaluation.

**Materials and Methods:**

This study used frozen semen straws from FH bulls selected according to criteria for proven (6 individuals) and unproven (6 individuals) bulls produced by the Singosari AI Center (AIC). Sperm motility parameters were assessed using Computer Assisted Semen Analysis (CASA Supervision^®^, Germany), while sperm viability and abnormality were evaluated through eosin-nigrosin staining under a microscope at 400´ magnifications. The integrity of the sperm plasma membrane was determined using the hypoosmotic swelling test, and acrosome integrity was analyzed using the fluorescein isothiocyanate PNA-propidium iodide staining method. Protamine deficiency was quantified using Chromomycin A3 fluorescence staining, while DNA fragmentation was assessed using the acridine orange technique.

**Results:**

The findings demonstrated that there were no statistically significant differences (*p* > 0.05) in the assessed parameters of frozen semen quality between FH-proven and unproven bulls. Furthermore, in FH-proven bulls, a negative correlation was observed between protamine deficiency and acrosome integrity (*r* = −0.528) and between protamine deficiency and sperm DNA fragmentation (*r* = −0.467). The parameters of protamine deficiency in unproven bulls exhibited a positive correlation with sperm progressive motility.

**Conclusion:**

The frozen semen quality of FH bulls in different classes (proven and unproven) was found to be equally good. Molecular-based analysis allows for a more accurate determination of semen quality. These findings are significant for bull breeding stations when comprehensively evaluating semen quality.

## Introduction

Enhancing dairy milk production can be achieved through improvement in nutritional management and the optimization of reproduction efficiency both in heifers and bulls; these factors can significantly impact their generational performance [1]. The evaluation of bull genetic characteristics about their generational performance and the selection of bulls for dairy production are conducted through progeny tests to determine their grade [2]. The selection of dairy cows based on grade aims to maintain livestock numbers and produce superior bulls. The National and Regional Artificial Insemination Center (AIC) in Indonesia has the function of producing and distributing superior quality and standardized frozen semen [3]. Dairy bulls at the Singosari AIC are classified into four categories: elite bull, proven sire, A grade, and B grade. The classification is based on the assessment of progeny tests conducted for each individual to determine the dairy bull’s productivity.

Bulls’ productivity is influenced by various factors, including genetic superiority, environmental [4], nutrition, management practices, and age [5]. One effective method to enhance the productivity of dairy bulls is the application of artificial insemination using frozen semen from superior bulls. The efforts to produce high-quality frozen semen are imperative for elevating livestock productivity and generating high-fertility offspring. The multiparametric measurement of frozen semen quality is related to freezing capability [6], which impacts the maintenance of sperm motility, normal morphology, intact acrosome, DNA integrity, and the functionality of sperm mitochondria, thereby influencing the fertilization process [7].

Semen quality is a sperm fertility-determining factor and can be assessed by macroscopic and microscopic. Conventional semen evaluation methods, which encompass macroscopic and microscopic techniques, are straightforward but may not yield entirely accurate results. The evaluation method to assess semen quality is developed using immunofluorescent techniques with fluorescent isothiocyanate peanuts-propidium iodide staining methods PNA-propidium iodide (determine acrosome status), acridine orange (AO) (DNA fragmentation level), and chromomycin (determine protamine deficiency) with a fluorescence microscope. The utilization of immunofluorescent techniques provides semen producers in Indonesia with a more precise molecular-based approach to determining frozen semen quality. Therefore, this study aims to assess the characteristics of frozen semen, providing valuable information and references for evaluating semen quality across different classifications. These findings are expected to contribute substantially to the development of improved reproductive management strategies in dairy farming. Additionally, the research findings hold significant relevance for AIs in Indonesia, underscoring the consistent similar semen quality between proven and unproven bulls. Such insights are pivotal for optimizing the utilization of semen from varied classes, thereby augmenting breeding programs and overall efficiency in dairy production.

## Materials and Methods

### Ethical approval

This study was conducted in accordance with the Indonesian National Standard (SNI ISO 9001:2015 No. G.01-ID0139-VIII-2019) at the Singosari AIC, under the supervision of a licensed veterinarian from Singosari AIC. Ethical approval for the research was granted by the Animal Care and Use Committee (reference No. 36/clearance/III/2021). Frozen semen samples were utilized in this study, with semen collected from healthy bulls using artificial vaginas without affecting the animal’s normal physiology.

### Experimental animals

Frozen semen straws from six bulls (proven and unproven bulls) were obtained from a one-batch production at Singosari AIC, East Jawa. These straws were analyzed to evaluate semen quality both before and following distribution to the breeding program, to identify any potential alterations in semen quality during the process.

Thawing of the frozen semen was performed by immersing the semen straws in a water bath at 37°C for 30 sec. The evaluation included multiple parameters, such as sperm motility, viability, abnormality, plasma membrane integrity, acrosome integrity, sperm DNA fragmentation, and protamine deficiency. All analyses were conducted at the Genomic Laboratory, National Research and Innovation Agency (BRIN), Bogor, Indonesia.

### Sperm motility, viability, and abnormality analysis

Sperm motility was analyzed using a computer-aided assessment system, the Sperm Vision Program (Minitub, Tiefenbach, Germany), in conjunction with a Carl Zeiss Microimaging GmbH (Göttingen, Germany), which was equipped with a warm stage maintained at 38°C. Aliquots of semen samples (5 μl) were placed onto a glass slide and covered with a coverslip. The kinematic parameters of approximately 7,500–10,000 sperm cells across five fields were evaluated using the Sperm Vision software, optimized for bull sperm analysis. The motility parameters assessed included total motility (TM) and progressive motility (PM).

Sperm viability and abnormality were evaluated using eosin-nigrosin staining. A 10 μl of semen was mixed with 20 μl of eosin-nigrosin staining solution, thoroughly homogenized, and smeared onto a glass slide. The smear was then dried on a heating table. Under a light microscope at 40× magnification, 200 sperm cells per slide were examined. Live sperm appeared with transparent heads, dead sperm with red-stained heads, and abnormalities (both primary and secondary) heads, dead sperm with red-stained heads, and abnormalities (both primary and secondary) were identified and recorded.

### Evaluation of sperm plasma membrane integrity, acrosome status, and sperm DNA fragmentation in frozen-thawed semen

Plasma membrane integrity was assessed using the hypoosmotic swelling test (HOS) test. A 20 μl semen sample was added to a microtube containing 300 μl of HOS solution (prepared with 0.735 gm Na citrate, 1,351 gm fructose, and 100 ml distilled water). The mixture was incubated in a water bath at 37°C for 30 min. After incubation, a drop of the semen-HOS solution was placed on a glass slide, covered with a coverslip, and examined under a light microscope at 40× magnification. Sperm with intact plasma membranes exhibited tail swelling, characterized by a bulging or circular shape. The percentage of sperm with intact membranes was calculated by analyzing at least 200 sperm cells, distinguishing those that reacted from those that did not.

The acrosome status of sperm was evaluated using the fluorescein isothiocyanate-peanut agglutinin (FITC-PNA) staining technique, following the protocol of Rajabi-Toustani et al. [8] with minor modifications. Sperm DNA damage was assessed using AO staining. A 20 μl aliquot of thawed semen was smeared onto a clean glass slide, air-dried, and fixed in Carnoy’s solution for 4 h. After fixation, the slide was rinsed with distilled water, air, dried, and immersed in AO solution in the dark for 12–15 h. The slide was then examined under a fluorescence microscope at 400× magnification with excitation light at 450–490 nm. Sperm with intact DNA emitted a green fluorescence, while those with fragmented DNA displayed yellow to orange fluorescence. DNA damage was quantified as a percentage by dividing the number of sperm with damaged DNA by the total sperm count and multiplying by 100%.

Sperm protamine deficiency was evaluated using Chromomycin A3 (CMA3), a fluorochrome that binds to the minor groove of DNA strands. Frozen semen from six bulls was thawed in a water bath at 37°C for 30 sec. Each semen sample was then washed in calcium- and magnesium-free phosphate-buffered saline and fixed in Carnoy’s solution (3:1 methanol to glacial acetic acid; Marck, Darmstadt, Germany).

### Statistical analysis

The data on frozen semen quality from Friesian Holstein (FH) bulls were analyzed descriptively. The means for sperm motility, viability, abnormality, intact plasma membrane integrity, acrosome status, DNA fragmentation, and protamine deficiency were analyzed using a one-way analysis of variance, with statistical significance *p *< 0.05. Correlations between the mean values of different bull classes and the quality of frozen semen were assessed using Pearson’s correlation test. All data analyses were performed using SPSS version 26 software (IBM^® ^Corp., NY).

## Results

The results indicated no statistically significant differences (*p* > 0.05) in frozen semen quality between proven and unproven FH bulls across all assessed parameters (Table 1). Likewise, the analysis of sperm motility patterns revealed no significant differences (*p* > 0.05) between the two classifications of FH bulls (Table 2).

**Table 1.** Post-thawing semen quality proven and unproven FH bulls.

Parameters	FH bulls	
	Proven ± SD (*n *= 6)	Unproven ± SD (*n *= 6)
Sperm motility (%)	58.2 ± 5.56	60.98 ± 4.44
Sperm viability (%)	72.09 ± 3.68	67.16 ± 12.54
Sperm abnormality (%)	4.77 ± 2.14	6.29 ± 2.06
Plasma membrane integrity (%)	68.7 ± 4.16	65.35 ± 8.47
Acrosome status (%)	96.65 ± 1.65	96.23 ± 2.30
DNA fragmentation (%)	6.23 ± 2.58	5.68 ± 2.24
Protamine deficiency (%)	2.66 ± 1.25	3.11 ± 1.36

**Table 2.** The sperm motility of post-thawed sperm in FH-proven and non-proven bulls.

Parameters	Grade FH bulls	
	Proven ± SD (*n *= 6)	Unproven ± SD (*n *= 6)
TM (%)	58.26 ± 5.56	60.98 ± 4.44
PM (%)	53.00 ± 6.59	55.03 ± 3.48
DAP (μm/sec)	36.89 ± 3.35	36.00 ± 2.84
DCL (μm/sec)	57.63 ± 6.68	57.87 ± 5.63
DSL (μm/sec)	21.20 ± 1.35	22.67 ± 1.26
VAP (μm/sec)	85.85 ± 6.53	81.57 ± 6.44
VCL (μm/sec)	132.02 ± 12.88	130.47 ± 12.03
VSL (μm/sec)	49.13 ± 2.33	51.64 ± 2.36
STR (%)	58.00 ± 6.16	63.33 ± 3.66
LIN (%)	37.16 ± 5.16	39.33 ± 2.33
WOB (%)	63.83 ± 2.31	62.33 ± 1.86
ALH (μm)	5.55 ± 0.34	5.07 ± 0.81
BCF (Hz)	26.28 ± 2.06	27.21 ± 1.44

TM (total motility); PM (progressive motility); DAP (distance average path); DCL (distance curve line); DSL (distance straight line); VAP (velocity of the average path); VCL (curvilinear velocity); VSL (velocity straight line); STR (straightness); LIN (mean linearity); WOB (wobble); ALH (amplitude of lateral head displacement); BCF (beat cross frequency).

The results indicated no statistically significant differences (*p* > 0.05) in distance average path (DAP), distance curve line (DCL), and distance straight line (DSL) between the two different classes of bulls (Table 2). Notably, the average values for PM average path velocity (VAP) (85.85 ± 6.53 μm/sec), curvilinear velocity (VCL) (132.02 ± 12.88 μm/sec), and straight-line velocity (VSL) (49.13 ± 2.33 μm/sec) were higher in proven FH bulls compared to unproven FH bulls, which had values of 81.57 ± 6.44, 130.47 ± 12.0, and 51.64 ± 2.36 μm/sec, respectively. Additionally, the average percentages of straightness (STR) and mean linearity (LIN) in unproven FH bulls were greater than those in proven FH bulls. However, these differences were not statistically significant (*p* > 0.05). The amplitude of lateral head displacement (ALH) and beat cross frequency (BCF) did not show significant differences between the classes of FH bulls (*p* > 0.05).

Furthermore, the analysis revealed no significant differences (*p* > 0.05) in sperm abnormalities, acrosome status, DNA fragmentation, and sperm protamine deficiency between the class of bulls (Table 1). A negative correlation was identified between protamine deficiency and acrosome status (−0.528) and protamine deficiency and DNA fragmentation (−0.467) in proven FH bulls (Table 3). Additionally, correlation analysis indicated a positive correlation between PM and protamine deficiency in unproven FH bulls (0.445) (Table 3).

## Discussion

Post-thawing sperm motility in both FH bull criteria (proven and unproven) stands in the normal range for insemination (average ≤40%). These studies were previously reported by Morrell et al. [9]. Sperm motility is a critical indicator of sperm quality and fertilization potential [10]. TM and PM are essential parameters in sperm motility assessment of frozen semen. These results align with those previously reported by Morrell et al. [9] in FH bulls.

The VAP and VCL values in this study were higher than those reported by Bahmid et al. [11], who reported that VAP and VCL values ranged from 39 to 75 μm/sec in FH bulls while remaining within the acceptable range for sperm motility. Diansyah et al. [12] reported that PM sperm exhibiting a VAP greater than 25 μm/s and progressive movement speed exceeding 20 μm/s could lead to successful fertilization.

No significant differences (Table 2) were observed in a DAP, DCL between the two classes of bulls. The average values for VAP (85.85 ± 6.53 μm/sec), VCL (132.02 ± 12.88 μm/sec), and VSL (49.13 ± 2.33 μm/sec) in proven FH bulls were greater than those in unproven FH bulls (81.57 ± 6.44 μm/sec, 130.47 ± 12.0 μm/sec, and 51.64 ± 2.36 μm/sec, respectively). The ability to fertilize is correlated with both velocity and LIN, which are essential characteristics of sperm fertility function [9].

The average wobble parameter (WOB) in proven FH bulls was higher than in un-proven FH bulls. According to Diansyah et al. [12], WOB is a measurement of periodic variation over time from a measurement result of the actual trajectory on the kinematic assessment of sperm movement. ALH and BCF are indicators of a sperm cell’s ability to survive in a sub-optimum environment [13]. In the hyperactivation group, three distinct patterns of sperm motility were identified: VCL >80 μm/sec, LIN < 65%, and ALH > 7 μm [14]. O’Meara et al. [15] reported the PM parameters of frozen semen ranged from 40% to 60%, while other parameters such as DAP were 25.97–31.64 (μm/sec), DCL 49.30–61.09 (μm/sec), DSL 16.58–20.60 (μm/sec), VAP 64.01–76.04 (μm/sec), VCL 121.10–146.14 (μm/sec), VSL 33.77–44.31 (μm/sec), STR 0.62–0.68, LIN 0.32–0.36, WOB 0.48–0.61, ALH 5.87–7.68 (μm), BCF 21.19–24.42 (Hz) [12]. Analyzing the patterns of frozen semen from proven and unproven FH bulls, particularly regarding parameters such as VAP, VCL, and ALH, showed promising results. Both criteria for these bulls exhibited favorable sperm fertility, as evidenced by adequate sperm motility. The parameters of VAP, VCL, and ALH indicate the potential for sperm hyperactivation penetration of the zona pellucida and are associated with elevated fertilization rates [16].

Sperm motility and viability are critical parameters for assessing the quality of a bull’s frozen semen. The results showed sperm viability in both groups of bulls met the criteria and demonstrated good quality. Morrell et al. [9] reported high sperm viability due to low reactive oxidative stress (ROS) formed during the semen freezing process. Low ROS is caused by using an antioxidant diluent during freezing to maintain the sperm membrane. In addition, low production of ROS in frozen semen sperm indicated metabolic activity decreased. Kowalczyk and Piatkowska [17] reported that high ROS levels during freezing could decrease motility and viability and increase sperm mid-piece abnormalities.

**Table 3.** Different bulls class correlation with frozen semen quality.

Bulls class		Prog. motility	Acrosome status	DNA fragmentation	Protamine deficiency
Proven	PM	1	0.171	−0.009	−0.038
	Acrosome status	0.171	1	0.253	−0.173
	DNA fragmentation	-0.009	0.253	1	−0.364
	Protamine deficiency	0.043	−0.528*	−0.467*	1
Unproven	PM	1	−0.100	0.398	0.455*
	Acrosome status	0.100	1	0.320	0.230
	DNA fragmentation	0.398	0.320	1	−0.244
	Protamine deficiency	0.455*	0.230	−0.244	1

*A significant correlation (*p *< 0.05).

The result showed that sperm abnormalities in both bull criteria are in the normal range (Table 1). Normal sperm morphology is related to lactate production in Sertoli cells, which helps prevent apoptosis during spermatogenesis [18]. Furthermore, the low rate of abnormalities may be linked to adequate testosterone production, which plays a role during the meiotic phase of germ cell development [19].

Both proven and unproven FH bulls exhibited comparable plasma membrane and acrosome integrity. Oliveira et al. [20] reported that the fertility rate of bulls is closely tied to the integrity of both the plasma membrane and the acrosome. However, it does not correlate with beef and dairy bull’s fertility index scores [10]. Decreasing the fertility of frozen sperm causes damage to cell membranes, which can stimulate ROS production by generating hydrogen peroxide and osmotic stress, reducing acrosome integrity [21], increasing DNA fragmentation, and protamine deficiency [22].

**Figure 1.**
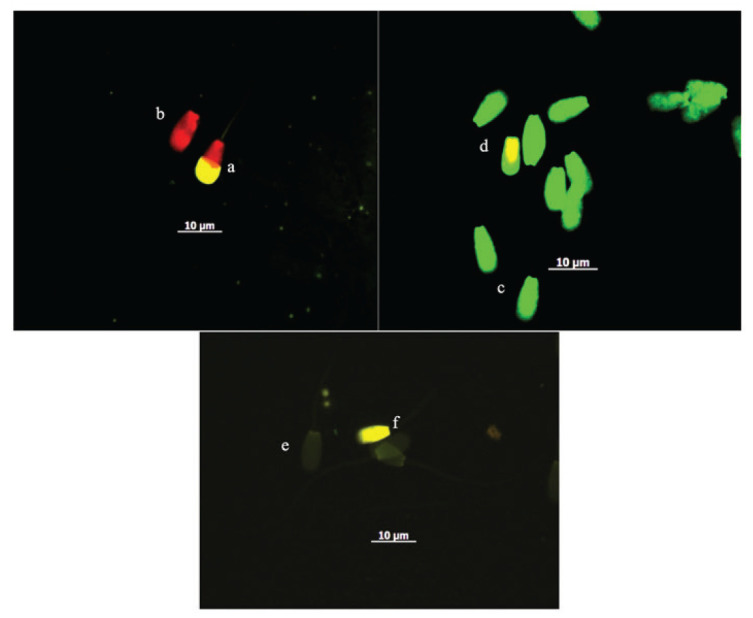
The sperm evaluation used fluorescence microscopy. Acrosome integrity was assessed using FITC-PNA with PI staining in FH sperm. Acrosome-intact sperm exhibited a bright yellow (a) over the acrosomal cup, and acrosome-loss sperm (b) showed no FITC-PNA staining; DNA fragmentation through AO destained shows that sperm displaying green (c) fluorescence were considered to be with normal DNA content, whereas sperm displaying a spectrum of green to yellow (d) fluorescence were considered to be with damaged DNA; sperm deficiency protamine using CMA3 staining. Yellowish green (e) round-headed sperm cells indicated protamine deficiency and bright yellow (f) round-headed sperm cells indicated normal protamine content.

The acrosome status and DNA fragmentation of sperm (Fig. 1) indicated that the production of ROS remained in equilibrium in accordance with the total antioxidant capacity of the cell during the freezing process [21]. Preserving this balance of ROS is essential for aerobic metabolism, membrane fluidity, and fertilization capability. It plays a critical role in capacitation and acrosome reaction by modulating the synthesis of cyclic adenosine monophosphate and tyrosine phosphorylation, thereby supporting sperm function during fertilization [23]. Furthermore, the two criteria for FH bulls in this study showed low protamine deficiency (Fig. 1), indicating their resilience to freezing. Carreira et al. [24] reported that bovine frozen sperm had lower protamine deficiency when compared with human sperm and resistance to cold stress temperatures.

The results revealed a negative correlation between protamine deficiency and both acrosome status and sperm DNA fragmentation in proven FH bulls (Table 3). Chromatin immaturity, which is linked to protamine deficiency, adversely impacts sperm functionally [21]. Additionally, the levels of protamine in both human and bull sperm, along with protamine deficiency, may be associated with DNA damage [24]. Moreover, freezing semen can affect protamine’s disulfide bonds, therefore increasing DNA damage and decreasing sperm acrosome status [24]. Sperm acrosome damage during the freezing process is under 50% because it will be affected by the ability of sperm fertility [25]. Furthermore, males in different cultures may exhibit varying levels of protamine deficiency. Michael et al. [26] reported that sperm DNA fragmentation below 15% is considered normal, while infertile males often have fragmentation levels exceeding 25%.

Sperm motility is a parameter that determines the sperm’s ability to fertilize the oocytes. PM parameter positively correlated with protamine deficiency in proven FH bull. Santoso et al. [27] reported that PRM1 expression was linked with PM in local Indonesian bull. Low PRM1 components can reduce PM by decreasing Ca2^+^ concentration, thus activating PRM phosphorylation and impacting protamine deficiency (PRM) [28]. Takeda et al. [29] reported that conditions in protamine deficiency are associated with mitochondrial damage, essential for flagella movement and sperm motility. The increase in DNA denaturation can trigger an apoptotic signaling pathway, leading to a decrease in mitochondrial function and subsequently reducing sperm motility [29].

The results of the correlation analysis showed a positive relationship between PM and protamine deficiency in unproven FH bulls (Table 3). The protamine deficiency effect can reduce sperm quality and fertility, including motility [27]. This study revealed a lower protamine deficiency level than that reported by Jalal et al. [30] using chromomycin staining and found a deficiency rate of 21.24%. Protamine, the primary nuclear protein in sperm, plays a crucial role in tightly packing sperm DNA, enhancing chromatin condensation, and safeguarding the genetic integrity of the paternal genome. Protamine deficiency in sperm affects the stability of sperm chromatin and impacts various morphological changes in the sperm head [7].

## Conclusion

FH bulls in different classes (proven and unproven) exhibited equally excellent quality frozen semen. Molecular-based semen analysis allows for a more precise determination of semen quality. These findings are valuable for breeding stations, facilitating a more thorough evaluation of semen quality in bulls.

## List of Abbreviations

ALH, amplitude of lateral head displacement; AO, acridine orange; ATP, adenosine triphosphate; AV, artificial vagina; BCF, beat cross frequency; DAP, distance average path; DCL, distance curve line; DSL, distance straight line; FH, Friesian Holstein; FITC-PNA, fluorescein isothiocyanate-peanut agglutinin; HOST, hypoosmotic swelling test; LIN, mean linearity; PM, progressive motility; PRM, protamine; ROS, reactive oxidative stress; STR, straightness; TM, total motility; VAP, velocity of the average path; VCL, curvilinear velocity; VSL, velocity straight line; WOB, wobble; ZP, zona pellucida.
